# Causality, Randomness, Intelligibility, and the Epistemology of the Cell

**DOI:** 10.2174/138920210791233072

**Published:** 2010-06

**Authors:** Edward R Dougherty, Michael L Bittner

**Affiliations:** 1 Department of Electrical and Computer Engineering, Texas A&M University, College Station, TX, USA; 2 Computational Biology Division, Translational Genomics Research Institute, Phoenix, AZ, USA; 3 Department of Bioinformatics and Computational Biology, University of Texas, M. D. Anderson Cancer Center, Houston, TX, USA

**Keywords:** Biology, causality, computational biology, epistemology, genomics, systems biology.

## Abstract

Because the basic unit of biology is the cell, biological knowledge is rooted in the epistemology of the cell, and because life is the salient characteristic of the cell, its epistemology must be centered on its livingness, not its constituent components. The organization and regulation of these components in the pursuit of life constitute the fundamental nature of the cell. Thus, regulation sits at the heart of biological knowledge of the cell and the extraordinary complexity of this regulation conditions the kind of knowledge that can be obtained, in particular, the representation and intelligibility of that knowledge. This paper is essentially split into two parts. The first part discusses the inadequacy of everyday intelligibility and intuition in science and the consequent need for scientific theories to be expressed mathematically without appeal to commonsense categories of understanding, such as causality. Having set the backdrop, the second part addresses biological knowledge. It briefly reviews modern scientific epistemology from a general perspective and then turns to the epistemology of the cell. In analogy with a multi-faceted factory, the cell utilizes a highly parallel distributed control system to maintain its organization and regulate its dynamical operation in the face of both internal and external changes. Hence, scientific knowledge is constituted by the mathematics of stochastic dynamical systems, which model the overall relational structure of the cell and how these structures evolve over time, stochasticity being a consequence of the need to ignore a large number of factors while modeling relatively few in an extremely complex environment.

## INTRODUCTION

As energy and matter lie at the basis of physical knowledge, the cell, as the basic unit of life, lies at the basis of biological knowledge and, therefore, ones epistemological stance towards the cell shapes ones understanding of the nature of biological knowledge. Since the epistemology of the cell must lie within in a wider scientific epistemology, scientific knowledge of the cell must satisfy the constraints applying to scientific knowledge in general. The intention of this paper is to articulate a properly biological epistemology of the cell, as opposed to merely viewing the cell as a product of many physical/chemical reactions without recognizing that these interactions must be regulated by the cellular components towards specific goal states that may be aimed at producing a cell locked into a particular differentiated state or shifting the cell to another differentiated state. This means viewing the cell as a system that fulfills the functions necessary for survival, such as regulation of protein production, communication among components within the cell and with extracellular components within the organism, information integration, response to external signals, self-organization in response to internal changes or external stimuli, and reproduction. 

Over the last seventy-five years, science and engineering have gained extensive experience modeling systems possessing these kinds of characteristics; indeed, we will formulate the epistemology of the cell in analogy with humanly constructed systems. In doing so we will address the two most fundamental epistemological questions arising relative to biology as a science: (1) What form does biological knowledge take? (2) How is biological knowledge validated? 

Before confronting these questions, we will direct our attention to a basic issue whose appreciation is prerequisite to developing a modern biology grounded on a sound scientific epistemology. One might refer to this issue as intelligibility. Are there categories of understanding that allow the formulation of biological science in terms that are intrinsic to Nature? Put another way, should we expect biology, as a manifestation of Nature, to be intelligible, in the sense that the mental models that constitute biological science make sense relative to our everyday interaction with natural phenomena? 

## PREDICTION IN BIOLOGICAL SYSTEMS

Biology became a discrete field of science with Darwin’s publication of *On the Origin of Species*, this being the first fairly comprehensive way of describing the characteristics that discriminate biology from chemistry and physics. Biology is the study of organisms, physical systems capable of retaining and utilizing information to execute processes that utilize available energy to organize matter for facilitation of their own persistence and reproduction. Reproduction can involve the passing on of slightly varied copies of the information as well as the combining of information from individuals possessing somewhat different sets of information. This constantly generated variance in organisms over time can produce different levels of fitness of the offspring organisms for particular environments. If the differences are sufficiently large to be a selective advantage, such variances can spread throughout the population of the organisms. 

The appearance of a theory of evolution in combination with a growing appreciation of the magnitude of the changes in extant organisms over time, through studies of the fossil record, served to focus attention on biology as a very long-running, continuous process. This in turn raised questions about how the information in organisms is coded and used to produce the range of capabilities within and across organisms. The clear adaptation of organisms to their environments raised questions about how the processes operating in biological systems are controlled to allow highly variable responses appropriate to both rapid and long-term changes in the environment. The persistence of organisms focused attention on how the extraordinarily complex biological processes required for an organism’s survival could be made sufficiently robust to account for long life-spans.

As in physics, the first types of relationships characterized in biology were ones where the process involved relied on simple, linear relationships. Study of the metabolic products common in organisms was an early and fruitful branch of chemical research, and by the beginning of the 1900’s, clear patterns of Mendelian heredity could be seen for diseases such as alcaptonuria, where the enzyme that catabolizes homogentisic acid is inactive and persons with the disease produce black urine, a result of the oxidation of excreted homogentisic acid. The general method of associating mutations of specific enzymes with failures to metabolize a particular substrate, biochemical genetics, was extremely successful in producing a clear understanding of the stepwise enzymatic manipulation of small molecules involved in anabolism, catabolism, and energy production. 

The prodigious success of biochemical genetics along with its very intuitive, easily understood methodology has deeply influenced how biologists think about and approach the study of biological processes. Metabolic processing relies on chains of enzymatic transformation of small molecules, where each step in the process is obligatory and each is typically carried out by a single catalytic entity. The processing is extremely efficient, with very little redundancy of activity and only modest branching and merging of the process chains. Much of the regulation of metabolic pathways is carried out at the level of the individual steps through feedback based on product levels, where either the amounts of enzyme made or the activity level of the enzyme are adjusted depending on changes in the concentration of its metabolic product. In analogy to physics, anywhere in biology that simple linear relationships are an appropriate approximation of the key interactions responsible for a particular phenomenon, model building is rapid and produces useful predictions that enable control. Unfortunately, biologists now face the barrier that confronted physics at the end of the 19^th^ century: most processes have sufficiently many conditioning influences that simple linear relationships cannot produce useful predictive models for them.

In facing the physicists’ dilemma, biologists will have to struggle with the same sorts of discomfort that the physics research community still endures. We possess a deeply rooted desire to visualize the relationships we are trying to characterize as simple relationships whose effects are easily intuited once a descriptive model is constructed. In physics, this desire can be best illustrated in the study of gravity. Newton’s model is very straightforward and can be thought of in a way that appeals to our own commonsense description of the world, namely, things fall as though attracted in some way to the earth. However, by 1900, there was sufficient experimental data to show that predictions made concerning Mercury’s orbital precession using this simple distance attenuated, attractive force model were sufficiently inaccurate to warrant reconsidering the model. Most effort was aimed at trying to find an explanation of the discrepancy that would fit within the Newtonian model. In the end, only a very different model, Einstein’s, was able to produce a better fit and make new predictions beyond the reach of the old model, such as gravitational lensing, and thus become the model of choice. In getting to this model, all of the comfortable assumptions about invariance in time and space had to be rejected, and as in quantum-mechanical physics, much of our universe became alien and non-intuitive. Recently, as physicists have been acquiring more and better data on larger scale objects, such as galaxies, discrepancies from the Einstein model have arisen and are provoking the same desires to find an accommodating explanation or produce a theory with a substantial difference. In the current phase, there are competing hypotheses, such as one that would remove the discrepancies by assuming that 94% of the universe is composed of “dark” matter that can only be sensed indirectly and one that would alter the way in which gravitational force changes over distance [1]. Whatever change eventually occurs will further distance us from our intuitive understandings. 

Acceptance of the need for models that will not provide biologists the simple and intuitive pictures of what they are studying will no doubt prove as difficult as it has been in physics. Even now, where it seems clear that the models physicists can produce will always be provisional, with new and different forms of data showing that the existing model only provides usefully accurate predictions for some restricted range of situations where further possible complications are not in play, there remain practitioners who expect there to be a simple grand unifying theory that will yet provide a simple and intuitive model of everything. Biologists, like physicists, must confront the issue of intelligibility. Owing to its special role in the ancient and medieval concept of the knowledge of Nature, causality plays a central role in our investigations, and that is where we begin. 

## CAUSALITY

The metaphysical character of causality has become ever clearer since the birth of modern science with Galileo. It plays no role in his scientific theories or in those of Isaac Newton. By 1913, Bertrand Russell could write, “The law of causality, I believe, like much that passes muster among philosophers, is a relic of a bygone age, surviving, like the monarchy, only because it is erroneously supposed to do no harm” [2]. Whereas Russell focused his criticism on the difficulty of defining causality meaningful to phenomena, a couple of decades later Erwin Schrodinger emphasizes the empirical vacuity of causality when he writes, “It can never be decided experimentally whether causality in Nature is ‘true’ or ‘untrue’. The relation of cause and effect, as Hume pointed out long ago, is not something that we find in Nature but is rather a characteristic of the way in which we regard Nature” [3]. Both Russell and Schrodinger follow in the wake of David Hume’s devastating criticism of causality of being neither logically nor empirically grounded. 

One would have thought the matter settled; however, it appears that Russell’s “relic of a bygone age” hangs on in biology. In a recent editorial entitled, “Biocomplexity as a Challenge for Biological Theory,” Werner Callebaut and Manfred Laubichler train their sights directly on Schrodinger when, discussing [4], they write, “Causation is still regarded here, with Hume and Kant, as ‘a characteristic of the way in which we regard Nature’ rather than intrinsic to Nature, which amounts to nothing less than renouncing knowledge of Nature itself” [5]. Not only do they assert causality but, in claiming that Hume, Kant, and Schrodinger renounce “knowledge of Nature itself,” argue for a return to pre-Galilean science since neither Galileo nor Newton required causal explanation for scientific knowledge. The statement is particularly perplexing because it is not made in reference to simple relationships that may admit deterministic modeling, but in the context of the extreme complexity confronting biological research, precisely the kind of massive-variable setting in which deterministic models fail most miserably. Looking back on three-quarters of a century of the success of modeling complex interacting phenomena using stochastic dynamical systems, it should be obvious that future success in biology depends on the application of such systems to the machinery of the cell. 

While Callebaut and Laubichler’s demand for causality is certainly a striking rejection of modern science in favor of a return to medieval science, it is not isolated. Even when questioning causality in biology, Ernst Mayr acknowledges causality at the level of the cell when he writes,

Most of the standard treatments of causality in the philosophical literature are based on problems in physics, where the effect of laws, such as those of gravity and thermodynamics may give an unambiguous answer to the question “What is the cause of…?’ However, such a simple solution is rarely available in biology except at the cellular-molecular level [6].

This is a strange statement because the question “What is the cause of…?” is never asked in physics, nor can it be asked in any scientifically meaningful way. Be that as it may, the notion that a “simple solution” based on causality could even be entertained in the extraordinary complexity of the cell simply ignores the last 100 years of science. 

Philosophers might speculate on some vague notion of causality underlying physical phenomena but such speculations are not part of science. In *Theory of Random Functions*, Vladimir Pugachev accepts the philosophical stance that phenomenological inter-dependence is a fundamental law of dialectical materialism, but then he goes on to explain the compatibility of that determinist metaphysical position with a stochastic scientific epistemology:

By virtue of this [law], each observable phenomenon is causally related to innumerable other phenomena and its pattern of development depends on a multiplicity of factors... Only a limited number of these factors can be established and traced. For this reason, if we observe the same phenomenon many times, it is seen that besides its general properties, there are certain special features which are only typical of a particular observation [7].

To wit, even under the assumption of determinism as a world view, successful modeling of complex phenomena requires stochasticity owing to the complexity. The assumption of a stochastic model is a scientific decision, not a metaphysical perspective. Andrei Kolmogorov, during the period when he was laying down the foundations of modern probability theory, states the matter concisely: “The possibility of using, in the treatment of a real process, schemes of well-determined or of only stochastically definite processes stands in no relation to the question whether the real process is itself determined or random” [8]. The so-called “real process” is not a subject of scientific knowledge. The question of whether cell function is deterministic or stochastic is not a scientific question and, even if cell function were deterministic, it would be highly unlikely that this determinism would be reflected in a gene network since the genes in the model would undoubtedly be affected by events (latent variables), including genes, outside the model, thereby imparting a stochastic nature to the model. This recognition is critical to the science of the cell and to translational science related to controlling cell behavior.

Having begun with some remarks about the present, let us go back and start from the beginning. In Book III of the *Physics*, Aristotle writes, “Knowledge is the object of our inquiry, and men do not think they know a thing till they have grasped the ‘why’ of it (which is to grasp its primary cause)” [9]. For Aristotle, causality has to do with providing categories of explanation. Knowledge is explanation surrounding the question of why and based on four causes, which, according to Aristotle, “perhaps exhausts the number of ways in which the term ‘cause’ is used”. A *material cause *is “that out of which a thing comes to be and persists”. It is “the bronze of the statue, the silver of the bowl, and the genera of which the bronze and the silver are species”. A formal cause is “the form or the archetype, i.e. the statement of the essence, and its genera… and the parts in the definition”. An efficient cause is “the primary source of the change or coming to rest; e. g. the man who gave advice is a cause, the father is the cause of the child, and generally what makes of what is made and what causes change of what is changed”. A final cause is “the end, or that for the sake of which a thing is done, e. g. health is the cause of walking about…. The same is true also of all the intermediate steps that are brought about through the action of something else as means toward the end”. Of the four causes, only Aristotle’s efficient cause is in accord with current common usage. For Aristotle, there is no clear demarcation between physics and metaphysics, so that the same causal categories are stated in both the *Physics* and the *Metaphysics*. 

In the *New Organon*, Francis Bacon agrees with Aristotle that causality is the ground of knowledge when he writes, “It is a correct position that true knowledge is knowledge by causes” [10]; however, he separates Aristotle’s four causes as to whether they apply to physics or metaphysics: material and efficient causes to physics, formal and final causes to metaphysics. But Bacon does not demarcate between science and metaphysics. While he sees no place for final causes in science, his preference for authentic scientific understanding lies with formal causes. In the *New Organon* he writes, “The efficient and the material (as they are investigated and received, that is, as remote causes, without reference to the latent process leading to the form) are but slight and superficial, and contribute little, if anything, to true and active science” [10]. Bacon separates physics from metaphysics, but it is within the domain of the latter where “true and active science” resides. Knowing the material out of which something comes to be or the source of change for a body’s change of motion is “superficial” in comparison to knowledge of form. 

Bacon desires a method to ascertain scientific truth based on experiment, not the abstract reasoning common in the medieval period or the anecdotal observations of ordinary experience. Given that true knowledge rests upon causality, then the form of knowledge and its acquirement should conform to causal relation. Thus, causality becomes inextricably linked to induction: when we observe that event *B* follows whenever event *A* is observed, then a cause-and-effect relation is in some sense logically induced between *A* and *B*. This relation goes beyond the list of observations to a deeper knowledge of reality. For Bacon, scientific knowledge is causal knowledge and this knowledge is reached by the “logical” process of induction upon observing one event, the effect, repeatedly following the other, the cause, without exception. Bacon recognizes that haphazard observation will not yield the kind of structured observations that lead to the discovery of inductive relationships. Therefore, he proposes that experiments be carried out in a ways to reveal sequences of events from which to induce causal relations. 

The relationship between causality and science takes a modern turn with Galileo, whose *Two New Science*s appeared in 1632, twelve years after the *New Organon*. Galileo recognizes that science concerns quantifiable relations among phenomena. He does not deny causality; rather, he sets the issue aside and gets on with pragmatic description. In *Two New Sciences*, Galileo puts these words into the mouth of Salviati:

The present does not seem to me to be an opportune time to enter into the investigation of the cause of the acceleration of natural motion, concerning which various philosophers have produced various opinions…. For the present, it suffices our Author that we understand him to want us to investigate and demonstrate some attributes of a motion so accelerated (whatever be the cause of its acceleration)…. [11].

In the terminology of phenomenology, Galileo *brackets* causality, ignores it, and gets on with the business of quantitative description. Although Galileo does not do away with causality, he rejects it as a requirement for knowledge.

In general, Galileo is dissatisfied with words. These constitute ersatz knowledge, the result being both an illusion of knowledge and an impediment to actual knowledge owing to satisfaction with empty phrases. When the Aristotelian Simplicio comments that everyone knows that bodies fall on account of gravity, Salviati responds,

You are wrong, Simplicio; you should say that everyone knows that it is called ‘gravity’. But I am not asking you for the name, but the essence of the thing. Of this you know not a bit more than you know the essence of the mover of the stars in gyration. We don’t really understand what principle or what power it is that moves a stone downwards, any more than we understand what moves it upwards after it has left the projector, or what moves the moon round [11].

Perhaps causality is operating here, but to simply say that there is a cause and to name it provides no knowledge. 

The issue of gravity comes to the fore when Isaac Newton formulates a mathematical law of gravitation that relates the distance, mass, and acceleration. The gravitational law is relational, mathematical, and idealized insofar as, when put into practice, it ignores confounding effects such as air resistance. It can be related to observations *via *experiment. The gravitational law mathematically characterizes a relation in such a way that the relation can be can used to make predictions, thereby providing a means for validation and for application. The mathematical structure represents a form of knowledge that is precise, inter-subjective, and operational. What it omits is any reference to some physical process behind the relation, in particular, to the cause of the acceleration. Russell writes, “In the motions of mutually gravitating bodies, there is nothing that can be called a cause, and nothing that can be called an effect; there is merely a formula” [2]. Like Galileo, Newton is not denying causality; he is bracketing it. Like Galileo, he is breaking with Aristotle and Bacon in formulating knowledge that does not depend on causality. Newton makes his intent clear In *The Principia: Mathematical Principles of Natural Philosophy*:

For I here design only to give a mathematical notion of these forces, without considering their physical causes and seats….It is enough that gravity does really exist, and acts according to the laws which we have explained, and abundantly serves to account for all the motions of the celestial bodies, and of our sea [12].

Newton recognizes that the need to limit oneself to a set of factors to arrive at mathematical representations of relations between phenomena imparts necessary limitations to scientific theories:

But our purpose is only to trace out the quantity and properties of this force from the phenomena, and to apply what we discover in some simple cases as principles, by which, in a mathematical way, we may estimate the effects thereof in more involved cases: for it would be endless and impossible to bring every particular to direct and immediate observation. We said, in a mathematical way, to avoid all questions about the nature or quality of this force [12].

The knowledge of which Newton speaks is mathematical, but it is not mathematics devoid of relation to human experience. It is empirically grounded, in that it facilitates prediction. The critical epistemological point is that the model is not meant to include all factors, but is of sufficient predictive power that it can “estimate” effects in a more general setting. 

When Galileo and Newton bracket causality, they begin a search for non-causal knowledge, thereby going beyond Aristotle and bringing about a radical change in epistemology; however, they do not come to grips with the meaning of causality. In particular, are a cause and its effect merely related *via *temporal priority, with the cause prior to the effect, or is there more than temporal contiguity? Is there a necessary connection between the cause and the effect? David Hume argues that in using the phrase “cause and effect” we mean the latter. In *An* *Enquiry Concerning Human Understanding*, he writes:

When one particular species of events has always, in all instances, been conjoined with another, we make no longer any scruple of foretelling one upon the appearance of the other, and of employing that reasoning, which alone can assure us of any matter of fact or existence. We then call one object, Cause; and the other, Effect. We suppose that there is some connexion between them; some power in the one, by which it infallibly produces the other, and operates with the greatest certainty and strongest necessity [13].

Does repeated observation of conjoined events warrant the supposition of a necessary connection? Is there a ground in reason or a physical ground for judging there to be a necessary connection? Hume states emphatically that there is no such ground. Belief in causality rests not on reason, but on habit. He writes,

But there is nothing in a number of instances, different from every single instance, which is supposed to be exactly similar; except only, that after a repetition of similar instances, the mind is carried by habit, upon the appearance of one event, to expect its usual attendant, and to believe that it will exist. This connexion, therefore, which we *feel *in the mind, this customary transition of the imagination from one object to its usual attendant, is the sentiment or impression from which we form the idea of power or necessary connexion. Nothing farther is in the case [13].

Repetition may lead to increased expectation, but not necessity – and certainly not to some deeper relationship. Induction does not depend upon causality; in fact, it is the opposite. Belief in causality is itself an unwarranted leap from repeated observations.

Modernity fully arrives with Hume. He does not bracket causality as a scientific category; he dismisses it as a scientific category by showing that it has no grounding in reason or in Nature, at least insofar as is empirically discernable. Necessary connections are subjective impressions, not objective relations. Observations lead to expectation, a probabilistic category, not to certainty. Scientific certitude is a fiction, a product of a leap of thought. Two centuries later, Schrodinger agrees with Hume and writes that “the relation of cause and effect is a characteristic of the way in which we regard Nature” [3].

Immanuel Kant also agrees with Hume that the principle of causality is not a product of reason. In the *Prolegomena to any Future Metaphysics*, he writes, “[Hume] justly maintains that we cannot comprehend by reason the possibility of causality, that is, of the reference of the existence of one thing to the existence of another, which is necessitated by the former” [14]. However, whereas for Hume, habit underlies belief in causality, for Kant, causality is a category of understanding. It is a form imposed on phenomena by the nature of the human mind. The mind imposes forms on the data of sensation, and scientific knowledge is limited by these forms. The way things appear, such as being spatially coordinated and connected by causality, are due to subjective *a priori* conditions for knowledge. One cannot know things apart from the manner in which they conform to these *a priori* mental forms. Of these categories of understanding, of which causality is one, Kant writes in the *Critique of Pure Reason*,

Now the question is whether there do not exist, *a priori* in the mind, conceptions of understanding also, as conditions under which alone something, if not intuited, is yet thought as object. If this question be answered in the affirmative, it follows that all empirical cognition of objects is necessarily conformable to such conceptions, since, if they are not presupposed, it is impossible that anything can be an object of experience. Now all experience contains, besides the intuition of the senses through which an object is given, a conception also of an object that is given in intuition. Accordingly, conceptions of objects in general must lie as *a priori* conditions at the foundation of all empirical cognition; and consequently, the objective validity of the categories, as *a priori* conceptions, will rest upon this, that experience (as far as regards the form of thought) is possible only by their means. For in that case they apply necessarily and a *priori* to objects of experience, because only through them can an object of experience be thought [15].

Only through the categories can an object of experience be thought. Hence, the mind’s structure imposes causality on our experiences as a prior condition for thinking about the experiences.

Kant’s argument imposes causality upon the phenomena we experience but not on the *things-in-themselves* that underlie the phenomena, the *noumena*, as he calls them. We cannot experience the things-in-themselves because they lie outside our sense experience. Kant asserts the existence of things-in-themselves, which for a strict empiricist like Hume cannot be asserted. Kant does not ascribe causality to the things-in-themselves, only to the phenomena we experience, and that because our minds impose causality on the phenomena as a condition of thinking about them. Whereas Galileo and Newton bracket causality, Kant moves it from Nature to the mind. 

From a scientific perspective, Kant takes a step backwards by making causality, not expectation, a category of understanding. Hume has already seen that the actual category of understanding is expectation. Observation of event *A* leads to the expectation of event *B*. Hume sees correctly that expectation is a probabilistic concept. There is no reason to raise the idea of causality. Why go beyond saying that upon observation of event *A* we expect to observe event *B*? 

Putting aside causality, Kant makes at least three key points regarding science. First, he insists that mind imposes human categories on the way in which Nature is understood. Experience does not arrive *qua* experience; rather, as human experience it arrives *via *the structure of the human mind. Kant puts mind, as an organizing and connecting entity, prior to experience. Second, he argues that, whatever ultimately lies behind the phenomena is outside the domain of science. A strict empiricist like Hume dogmatically asserts that one cannot speak of anything lying behind the phenomena. Kant argues otherwise and, in doing so, is more in line with Newton; who believes that gravity exists, although he can say nothing about it except what is revealed by the mathematical formulae expressing phenomenal relations. Third, Kant contends that science is a product of the human mind and, because science is limited by its epistemology, the mind is only bound to the conclusions of science when it operates within the categories of understanding, which themselves are limited to phenomenal experience, and therefore are not operative outside the domain of that experience. This is a strong statement regarding limitations of human understanding because it says that the categories of understanding, which apply to phenomena, cannot be legitimately applied outside phenomena. Metaphysics is not ruled out, but generalizing phenomenal categories to metaphysics is not legitimate. 

Whereas Kant sees Hume’s arguments concerning the lack of empirical ground for causality as definitive, the empiricist John Stuart Mill wishes to empirically ground science in the aftermath of Hume, which, for him, means grounding induction and, in turn, causality. In *A System of Logic, Ratiocinative and Inductive*, he gives his definition:

The Law of Causation, the recognition of which is the main pillar of inductive science, is but the familiar truth that invariability of succession is found by observation to obtain between every fact in nature and some other fact which has preceded it, independently of all considerations respecting the ultimate mode of production of phenomena, and of every other question regarding the nature of ‘Things in themselves’ [16].

There are four fundamental points regarding Mill’s view: (1) no necessary connection is implied by causality; (2) the effect must be the “invariably and unconditionally consequent” of the cause; (3) causality makes no reference to what is behind the phenomena; and (4) causality is “coextensive with human experience.” Mill escapes Hume’s criticism by abandoning any notion of necessary connection and making induction purely sequential, but misses Hume’s salient scientific point, the impossibility of arriving at the unconditional invariability of succession by any finite number of observations. 

Mill recognizes that the causality cannot be as simple as that of a single event being the sole cause of an effect. Regarding the complexity of causation, he writes,

But the real cause is the whole of the antecedents, the whole of the contingencies of every description, which being realized, the consequent invariably follows. Yet even invariable sequence is not synonymous with causation. The sequence, besides being invariable, must be unconditional [16].

Clearly, “the whole of the antecedents, the whole of the contingencies of every description” has no bounds and may very well be the entire universe, which would reduce the entire notion of cause and effect to a statement about universal determinism, a position taken by Mill. But if causality depends on knowing all the antecedents composing a cause, then surely it is not coextensive with human experience. On the other hand, expectation is very much coextensive with human experience. 

Mill recognizes that, when applying induction in the course of scientific discovery, haphazard observation will not do. He writes, “Experimentation has great advantages over observation in that it often enables us to obtain innumerable combinations of circumstances which are not to be found in nature” [16]. But instead of the Galilean-Newtonian recognition that experimental constraint leads to relations that “estimate” relations among naturally occurring phenomena, Mill wants to use experiment to obtain “innumerable combinations of circumstances”, a goal that on its face is impossible. 

In trying to circumvent Hume’s attack on causality on strictly empiricist grounds, Mill returns to a pre-Galilean world in the sense that, although necessary connection is abjured, causality remains a requirement for knowledge. Hume’s analysis regarding uncertainty and the impossibility of concluding a necessary connection, one that is unconditional and invariable, is impenetrable because the certainty of formal logic does not apply to human interaction with Nature. Expectation, not causality, is coextensive with human experience. 

In his essay, “On the Notion of Cause”, Russell demonstrates the impossibility of giving precise meaning to several different attempts to define “cause”. He settles on the previously cited definition of Mill as perhaps the best attempt at a viable definition: “The Law of Causation, the recognition of which is the main pillar of inductive science, is but the familiar truth that invariability of succession is found by observation to obtain between every fact in nature and some other fact which has preceded it” [16]. But this attempt fails owing to the impossibility of supplying it with a suitable notion of event and the “insuperable difficulties”, which Russell carefully articulates, of trying to define the timing between a cause and an effect. Recognizing that Mill’s reasoning regarding induction and causality are based on the appearance of uniformities in nature, Russell acknowledges such, even to the possibility that no exception has ever been witnessed. Nonetheless, he writes,

What I deny is that science assumes the existence of invariable uniformities of sequence of this kind, or that it aims at discovering them. All such uniformities, as we saw, depend upon a certain vagueness in the definition of the ‘events’… The principle ‘same cause, same effect,’ which philosophers imagine to be vital to science, is therefore utterly otiose. As soon as the antecedents have been given sufficiently fully to enable the consequent to be calculated with some exactitude, the antecedents have become so complicated that it is very unlikely they will ever recur. Hence, if this were the principle involved, science would remain utterly sterile [2].

Hume’s analysis marks the end of any hope that science should involve certainty, or unconditional and invariable sequences. In the *Rise of Scientific Philosophy*, Hans Reichenbach writes,

Empiricism broke down under Hume’s criticism of induction, because it had not freed itself from a fundamental rationalist postulate, the postulate that all knowledge must be demonstrable as true. For this conception the inductive method is unjustifiable, since there is no proof that it will lead to true conclusions [17].

Science does not depend on unconditional sequences. One need not turn to physics to see this; it is more readily recognized in biology, where the subject matter begins with the cell, whose behavior is conceptualized as a random dynamical process. This does not mean that science is ungrounded, only that it must be grounded in probability theory, not in deterministic logic.

## RANDOMNESS

Whereas Hume properly placed knowledge with respect to observed regularities into the domain of probability and since we wish to consider biological knowledge in a stochastic framework, we need to address another troublesome word: “random”. All too often the word “random” is tossed about without heed to a rigorous definition. For instance, the time between two events, such as between completion of transcription to initiation of translation, is said to be random, or the occurrence of an event, such as a specific gene mutation, may be described as random. But do expressions such as these have meaning? That depends on the possibility of defining the notion of randomness.

Webster’s Unabridged Dictionary defines the adjective “random” as, “without aim or purpose; haphazard” [18]. Purpose is defined in two ways. The first is “that which a person sets before himself as an object to be reached or accomplished; aim; intention; design”. Under this definition, a random occurrence is one that is not intended or designed. For the two aforementioned biological examples, this would say that there is no intended or designed time between the completion of transcription and the initiation of translation, and that mutations are neither intended nor designed. This is clearly not what is meant by “random” in science because science, as an empirically based discipline, is not concerned with the intention of beings situated outside the phenomena. The second Webster definition of purpose is an “end in view; the object for which something exists or is done”. Under this definition, a random occurrence is one without object. For our two examples, we see no way of making this definition relevant to the timing between transcription and translation; however, it could be applied to say that mutations occur without an end in view. But this latter interpretation puts us back into the domain of causality because it says that mutations occur without final cause and even Bacon considers final causality as part of metaphysics. 

Although we cannot appeal to Webster’s Dictionary for a definition of randomness insofar as science is concerned, the relation to final cause opens the door to an alternative view of randomness, that a random event is one not determined by causality. Here there is a mixture of two concepts, determinism and causality. Let us first dismiss causality because if a random event were defined as a non-causal event, then randomness would be defined as a negation of a nonscientific category and therefore would, itself, be nonscientific. Hence, we arrive at a random event being one that is not determined. But this throws us back upon determinism as a category pertaining to phenomena, absent a notion of causality; however, determinism is meaningless as a scientific category because the hypothesis of determinism is phenomenally vacuous. Thus, if randomness is the negation of phenomenal determinism, then it, too, is meaningless.

Randomness is indeed related to determinism, not in terms of phenomena but rather in how we treat phenomena within science. Since science is about relations, scientific knowledge involves the representation of relations. Since scientific sensibility involves measurements, either logical or numerical, scientific knowledge concerns relations between measurements. Finally, because we are interested measurements of recurring phenomena, the objects to be related take the form of abstract symbols, called “variables”, that represent measurements, such as distance, time, protein abundance, etc. An example is the time *r* between transcription and translation. For a specific instance, *r* represents a single measurement and takes the mathematical form of a real number. However, our interest is not with a single observation of time but rather the class of measurements. Thus, the time between transcription and translation varies depending on a host of conditions within the cell and the time is represented as a random variable, which we will denote by *R*. Here, the word “random” appears as part of the term “random variable”, which has a precise mathematical definition. The nature of the random variable *R* is more subtle than that of a simple real number and it was not until the twentieth century that we had a suitable definition of a random variable, that being a “measurable function” from a “probability space” into the space of real numbers. This definition requires the definitions of a measurable function and a probability space, which in turn requires the definition of a probability measure, the upshot being that it requires the development of the mathematical theory of measure to be able to give meaning to the word “random” in regard to its scientific usage. 

Let us extend this simple case to one more representative of a biological system. Consider a dynamical process, say the amount of the protein product, Wnt5a, corresponding to the gene WNT5A, in a cell. Dynamically, this measurement is represented as a variable of the form *x*(*t*), where *t* denotes time and *x*(*t*) is a numerical value whose units depend upon the measurement procedure. If we track this abundance for a single cell, we get a time function that is deterministic, the latter meaning, by definition, that there is a certain value at each time point. However, we are typically interested in the behavior of Wnt5a for an arbitrary cell and, then, the measurement is not deterministic, the abundance trajectory being different for different cells. In this case the measurement is represented as a time-dependent random variable, denoted *X*(*t*), again the word “random” appearing as part of the mathematical term “random variable”. The deterministic variable *x*(*t*) takes values in some numerical space, such as the logical space {0, 1}, the integers, or the real line, depending on the quantization of the measurement procedure. The random variable *X*(*t*) is a function from a probability space into a numerical space. Whereas as *x*(*t*) is referred to as a “ time function”, *X*(*t*) is referred to as a “random time function”, a “random time process”, or a “stochastic process”. In every instance the word “random” is used, it requires a definition in terms of the underlying mathematical spaces. None of this makes any suppositions concerning things-in-themselves. In the context of science, “random” is simply a word adopted by mathematics and defined therein within the framework of axiomatic probability theory. 

To see what happens when one tries to use the word “random” loosely, consider the following statement by Francisco Ayala:

Mutations are said to be accidental, undirected, random, or chance events. These terms are often used as synonyms, but there are at least three different senses in which they are predicated of the mutation process. First, mutations are accidental or chance events, in the sense that they are rare exceptions to the regularity of the process of DNA replication, which normally involves precise copying of the hereditary information, encoded in the nucleotide sequences. Second, mutations are accidental, random, or chance events also because there is no way of knowing whether a given gene or genome will mutate in a particular cell or in a particular generation. We cannot predict which individuals will have a new mutation and which ones will not, nor can we predict which gene will mutate in a given individual. This does not imply that no regularities exist in the mutation process; the regularities are associated with stochastic processes, to which probabilities can be assigned. There is a definite probability (although it may not have been ascertained) that a given gene will mutate in any given individual. Moreover, it is not true that a mutation is just as likely to occur as any other mutation. Third, mutations are accidental, undirected, random, or chance events in a sense that is very important for evolution; they are unoriented with respect to adaptation [19].

Note the terms Ayala is grouping with “random”. Both “accidental” and “undirected” agree with Webster because they relate to unintended events. So it seems that he is in agreement with Webster and at the outset leaves the domain of science for psychology or metaphysics. Yet his first definition does not speak of intent; rather, he uses randomness to describe a process that exhibits rare exceptions to regularity, hence, seeming to imply that randomness applies to a non-causal process, in the sense of Mill, thereby making it, at best, a metaphysical category or, at worst, meaningless. Skipping momentarily to the third definition, this defines random as being non-causal in the sense of final cause, again metaphysical. The second definition is the only one suitable for science. Although he does not go into a careful mathematical characterization of mutations forming a stochastic process, one can be given. Ayala’s contention that all three definitions are used in biology is where the problem lies. The first and third have nothing to do with science, so that their use pollutes biological knowledge – the result of a lack of attention to sound epistemology. 

## INTELLIGIBILITY

When Newton writes, “And to us it is enough that gravity does really exist”, he is bracketing causality along with whatever “physical” substance is represented by the phenomena observed. What perhaps Newton did not realize is that this bracketing would become permanent in the sense that today there is no explanation of gravitation as a physical substance; indeed, one is hard pressed to say what is meant by a “physical substance”. What is certain, however, is that the Newtonian gravitational law and the more modern theories in terms of the curvature of space are mathematically clear and make excellent predictions. When discussing the enormity of the transformation wrought by Galileo and Newton, Morris Kline writes, “What science has done, then, is to sacrifice physical intelligibility for the sake of mathematical description and mathematical prediction” [20]. Sacrificing physical intelligibility does not involve an abandonment of knowledge; on the contrary, it involves the recognition that everyday human categories concerning Nature – those that arise from the ordinary interaction with the physical world, such as pushing and pulling – are not suitable for describing phenomenal relations. Kline goes on to say,

The insurgent seventeenth century found a qualitative world whose study was aided by mathematical abstractions. It bequeathed a mathematical, quantitative world that subsumed under its mathematical laws the concreteness of the physical world. In Newton’s time and for two hundred years afterwards, physicists spoke of the action of gravity as ‘action at a distance’, a meaningless phrase that was accepted as a substitute for explaining the physical mechanism, much as we speak of spirits or ghosts to explain unseen phenomena [20].

Consider the electromagnetic field theory that is responsible for so much technology in the modern world. The theory, rooted in James Clerk Maxwell’s equations, is completely understood because it is a mathematical theory. Its applications depend on the behavior of detectors as predicted by the theory. But what is the nature of the physical substance behind these? In *On Faraday’s Lines of Force*, Maxwell explains how he will analogize lines of force as “fine tubes of variable section carrying an incompressible fluid”. He writes,

I propose, then, first to describe a method by which the motion of such a fluid can be clearly conceived; secondly to trace the consequences of assuming certain conditions of motion, and to point out the application of the method to some of the less complicated phenomena of electricity, magnetism, and galvanism; and lastly to shew how by an extension of these methods, and the introduction of another idea due to Faraday, the laws of the attractions and inductive actions of magnets and currents may be clearly conceived, without making any assumptions as to the physical nature of electricity, or adding anything to that which has been already proved by experiment. By referring everything to the purely geometrical idea of the motion of an imaginary fluid, I hope to attain generality and precision, and to avoid the dangers arising from a premature theory professing to explain the cause of the phenomena [21].

The key point is that Maxwell obtains his theory “without making any assumptions as to the physical nature of electricity”. 

Although the theory is fully consistent with experimental measurements, Maxwell is not fully satisfied because the theory lacks physical intelligibility. He continues,

If the results of mere speculation which I have collected are found to be of any use to experimental philosophers, in arranging and interpreting their results, they will have served their purpose, and a mature theory, in which physical facts will be physically explained, will be formed by those who by interrogating Nature herself can obtain the only true solution of the questions which the mathematical theory suggests [21].

Maxell’s dissatisfaction arises from the fact that he cannot explain the theory in purely physical categories. Where does the matter sit today? Kline writes:

We do not have any physical account of the knowledge of the electromagnetic waves as waves. Only when we introduce conductors such as radio antennae in electromagnetic fields do we obtain any evidence that those fields exist. Yet we send radio waves bearing complex messages thousands of miles. Just what substance travels through space we do not know [20].

As Newton brackets causality and the physical nature of gravity in favor of mathematical relations, Maxwell brackets the physical waves behind the field theory. In general, science foregoes physical intelligibility (in the standard Aristotelian sense, which is the everyday sense), and constitutes knowledge within mathematical structures that allow us to build devices that respond according to the equations and thereby produce pragmatic effects in the physical world that are in some unknown way a result of the bracketed waves.

Perhaps there is dissatisfaction, a nostalgic yearning for the simple Aristotelian notion that the mind can grab hold of reality. On this account Kline remarks,

Our mental constructions have outrun our intuitive and sense perceptions. In both theories, gravitational and electromagnetism, we must confess our ignorance of the basic mechanisms and leave the task of representing what we know to mathematics. We may lose pride in making this confession, but we may gain understanding of the true state of affairs [20].

Kline’s use of the word “understanding” is interesting. We understand the mathematics, and not the things-in-themselves, but because the predictions from the mathematics relate the mathematics to the phenomena, we are closer to the true state of affairs, whether in the Humean sense that the phenomena are all of which we can speak or in the Kantian sense that there are noumena truly existing behind the phenomena.

## SCIENTIFIC KNOWLEDGE

We have discussed the elimination from science of causality and other physically intuitive notions and traced the evolution of scientific epistemology through Maxwell. We believe this is sufficient to have established the basic empirical-mathematical duality underlying scientific knowledge, which we now briefly discuss (see [17] for a thorough treatment of modern scientific epistemology and [4] for a treatment aimed at computational biology).

Scientific knowledge necessarily takes the form of mathematics for four reasons: (1) scientific knowledge is based on quantitative measurements, be they logical or numeric; (2) scientific knowledge concerns relations and mathematics provides the formal structure for relations; (3) the validity of a scientific theory depends on predictions and this requires a quantitative structure from which to generate predictions and a theory of probability in which the goodness of predictions can be quantified; and (4) mathematics provides a formal language sufficiently simple so that both the constituting theory and the experimental protocols for prediction are inter-subjective, once the underlying mathematical representation of the theory is agreed upon. A theory does not stop at the defining relations; it includes propositions deduced from the defining relations and this deduction can reveal critical relations not at once apparent in the defining relations. A full mathematical model consists of the defining relations and all relations logically deduced from these.

While the form of scientific knowledge is mathematical, that alone does not provide scientific knowledge because scientific knowledge must have an empirical base. Moreover, scientific knowledge is poorly served by anecdotal observations; instead it should rely on structured experiments. In regard to the movement from unplanned observation to probing Nature according to a structured protocol to elicit observations directly aimed at constructing or validating a set of mathematical relations, Kant asserted, “To this single idea must the revolution be ascribed, by which, after *groping in the dark* for so many centuries, natural science was at length conducted into the path of certain progress” [15]. The product of an experiment is a set of measurements. These form the data of sensibility, the empirical (as opposed to a rational) basis for knowledge.

Where is the mathematical model to come from and how does one characterize model validity relative to a measurement process? According to Albert Einstein, the mathematical model, or as he called it, the “conceptual system” is a creation of the “imagination”. The manner of this creation is not part of the scientific theory. The classical manner is that the scientist combines an appreciation of the problem with reflections upon relevant phenomena and, based upon mathematical knowledge, creates a model. The model arises from empirical considerations. But the most critical aspect of the relationship between the model and phenomena occurs in the validation process because it is the validation process that characterizes scientific “truth”. 

Recalling Hume, regularity in structured observations leads to expectation. Thus, there is no truth in the sense of logical certainty; scientific truthfulness lies in prediction. As Reichenbach states, “Scientific philosophy has constructed a *functional* conception of knowledge, which regards knowledge as an instrument of prediction and for which sense observation is the only admissible criterion of nonempty truth” [17]. A model’s formal structure must lead to experimental predictions in the sense that there are relations between model variables and observable phenomena such that experimental observations are in accord with the predicted values of corresponding variables. There must be a predictive framework for validation because the scientific truth, or validity, of the model depends on the accuracy of predictions arising from the model. This requires the model to be related to the experimental methodology. Reichenbach states, “The reference to verifiability is a necessary constituent of the theory of meaning. A sentence the truth of which cannot be determined from possible observations is meaningless” [17]. Verification of a system requires that the symbols be tied to observations by some semantic rules that relate not necessarily to the general principles of the mathematical model themselves but to conclusions drawn from the principles. In other words, the theory is tested by checking measurable consequences of the theory. These *operational definitions*, as they are called, are an intrinsic part of the theory, for without them there would be no connection between the principles and observation. There must be a well-defined procedure for relating the consequences of the equations to quantifiable observations, such as gene expression in the steady state of a gene regulatory network. A scientific theory must have two parts: a structural model and a set of operational definitions for its symbols.

A great power of the scientific epistemology lies in the logical deducibility of logically necessary relations from the relations defining the model – the *hypothetico-deductive method*. The knowledge constituted by these derived relations is implicit in the defining mathematical system but only becomes apparent when derived explicitly. Often, the most striking aspects of a scientific theory are represented by these derived relations – for instance, the consequences of Newton’s gravitational law and of Maxwell’s equations. In particular, key applications are typically the result of consequences of the basic model. Moreover, as just noted, model validation is achieved *via *predictions made from the consequences.

The preceding prescription does not lead to a unique, absolute truth because validation is a process and the “truth” of the theory is relative to that process. Einstein states, “In order that thinking might not degenerate into ‘metaphysics’, or into empty talk, it is only necessary that enough propositions of the conceptual system be firmly enough connected with sensory experiences” [22]. What is left unclear in Einstein’s statement is a precise definition of what is meant by “enough propositions” and what it means to be “firmly enough connected with sensory experiences”. Operational definitions are required, but their exact formulation in a given circumstance is left open. Their specification constitutes an epistemological issue that must be addressed in mathematical (including logical) statements. Absent such a specification, a theory is meaningless. Because a model consists of mathematical relations and system variables must be checked against quantitative observations, there is no nonmathematical way to describe the requirements and protocols to assess model validity. Hence, mathematics is essential to the structure of the model and its verification.

Perhaps the lack of absolute and unique knowledge is unsettling to some, as it has been to some of the great minds of science. One could take solace in the bracketing of Galileo and Newton just because the search for physical truth was bracketed and not abandoned. Hume, however, represents a coming of age, and from him there is no return – at least if one is going to be serious. The critical philosophy of Kant was a reaction to Hume but Kant offers little solace to one who wants to know physical truth *qua* Nature. Modern physics has only accentuated this and, as we will shortly discuss, biology drives home the point even further. Let us close this section with a quote from Einstein:

Physical concepts are free creations of the human mind, and are not, however it may seem, uniquely determined by the external world. In our endeavor to understand reality we are somewhat like a man trying to understand the mechanism of a closed watch. He sees the face and the moving hands, even hears it ticking, but he has no way of opening the case. If he is ingenious he may form some picture of the mechanism which could be responsible for all the things he observes, but he may never be quite sure his picture is the only one which could explain his observations. He will never be able to compare his picture with the real mechanism and he cannot even imagine the possibility of the meaning of such a comparison [23].

Kant might have written these words in regard to the futility of a scientific search for the things-in-themselves. 

## EPISTEMOLOGY OF THE CELL

Wilhelm Windelband defines epistemology in the following way: “The problems, finally, which arise from the questions concerning the range and limit of man’s knowing faculty and its relation to the reality to be known form the subject-matter of epistemology or theory of knowledge” [24]. We take the word “range” to refer to the kind, or nature, of the knowledge under consideration, many definitions of epistemology referring to the nature of knowledge. Thus far, our discussion has been on the epistemology of science, in particular, the nature and limits of scientific knowledge, as well as its relation to reality. This relation is *via *observations of the phenomena that result from the things-in-themselves – and in that sense we take a Kantian position that there are things-in-themselves outside the direct structure of our scientific knowledge, which is constrained by the limits of sensibility. The observations are tied to scientific knowledge, which is constituted by mathematics, and the validity of the knowledge is relative to predictions based upon the mathematics and tested *via *operational definitions. All of this is general, applying to any branch of science. The epistemology of biology has its own unique issues and, therefore, unique nature within the general scientific epistemology. Biology is not physics, nor is it chemistry. Our problem is the nature of biological knowledge, in particular, biological knowledge of the cell, as opposed to general physical or chemical knowledge of the cell.

Biology concerns living organisms. These exist in the physical world. Therefore biology depends upon physics. Each cell consists of a host of molecules that form the building blocks of structures within the cell and are involved with interactions both interior and exterior to the cell. Therefore biology depends upon chemistry. But the subject matter of biology is not that of physics or chemistry; otherwise, biology would be a branch of physics or chemistry. Biology concerns the operation of the cell in its pursuit of life, not the molecular infrastructure that forms the physiochemical underpinnings of life. The activity within a cell is much like that within a factory. In the latter, machines manufacture products, energy is consumed, information is stored, information is processed, decisions are made, and signals are sent to maintain proper factory organization and operation. All of these functions also take place within a cell and it is through analogy with a factory that we approach the epistemology of the cell.

The hardware units within a factory, whether mechanical, electrical, or chemical, do not constitute the factory. These require specialized knowledge to build and are necessary for the factory to function but, in and of themselves, they simply compose a collection. They become part of a factory when their functioning is organized and regulated according to a logical program that integrates and orders their activities in such a way as to produce the desired products and maintain their proper functioning within the overall operation of the factory. If we strip away all of the components – the robots, the computers, the communication devices, the relays, etc. – that is, the units within the factory that could be individually used for any number of purposes, what remains, and what constitutes the factory as an entity, is the regulatory logic that controls the dynamics of the factory. 

The same can be said for a cell if we strip away that which is purely physical and chemical. While it is true that transcription factors are required to implement the regulatory cell logic, the chemical interactions involved in the functioning of the transcription factors are a subject for chemistry, in the same way that the electrical impulses that carry the instructions to robots in a factory are a subject for physics. One can know all of these reactions but be no closer to understanding the livingness of the cell. One can list a multitude of the interactions between molecules within the cell, as one could write down the entire instruction set of a computer, but without the program that regulates the manner in which the instructions are used, there are only the symbols of codes, not functioning codes that convey information. 

A first and necessary step for modeling cellular processes and their regulation is, therefore, to begin to consider what level of regulation would be a useful target of study. This consideration is a form of detail triage that must be applied as a consequence of the considerable complexity in some types of cellular regulation. At the simplest level of regulation, the core functions of metabolism deal with the most basic and ubiquitous functions required for the cell to be able to carry out any further function. As would be expected for functions that have been continuously selected for continuity of operation and maximal efficiency for as long as organisms have existed, their regulation is tuned to maximize the operational utility of individual steps. There are some cases where these processes may need a large regulatory adjustment, such as hypoxia, or scarcity of an exogenously supplied carbon source used to derive energy or construct macromolecules. Yet for the most part, adaptations of the processes to variation in source materials and requirements for energy on the input side and energy expenditure and molecular construction on the output side fall well within the capability of adjustments determined at the local level. 

There are critical issues that a well-run factory must confront, each with an analog within the cell. A factory, or computer system, must handle interrupts. A factory is not a closed system. It has inputs and outputs, and it also has unplanned emergencies, such as the failure of some component, the loss of its primary energy supply, or a change in the demand for its products. Interrupts do not occur with fixed regularity, and in this sense every input can be considered an interrupt because the timing of input arrivals cannot be synchronously regulated. In order that the factory not completely shut down for an extended time, which could result in economic failure, its operational program must have procedures for redirecting the activities within the factory to handle the interrupt until such time that the effects of the interrupt have passed and the factory can return to normal function, a sort of homeostasis. Consequently, a factory’s activities must be modeled stochastically. Basically, while all nodes within the factory may appear to function with complete regularity under “normal” functioning, in fact, the factory is affected by many latent variables unaccounted for no matter how complex the model and these give the factory a stochastic character. 

Cells have a similar approach to managing their responses to critical changes that could lead to death or substantial damage to the organism. These actions fall in the category of stress responses, system-wide alterations that deal with various environmental insults and cellular malfunctions. In these situations, many processes may need to be halted and many others instituted. A familiar example of this kind of regulation is the response to damage from ionizing radiation. At this level of regulation, the concept of cellular context becomes evident. An organism has many different kinds of cells and reacts to damage to them in quite different ways. Cells that produce the precursors of short-lived cells needing frequent replacement, such as blood cells or cells that line the gut, are typically much more likely to invoke a death response when their DNA is damaged than cell types like neurons, which are very slow to replicate. The same stimulus is interpreted in different ways in these cells even though the mechanism of recognizing the damage is the same. In these cases, the interpretation of the recognition of damage is conditioned by interactions with different genes present in the different cell types. In regulatory processes, where chains of signals are used to induce systemic changes in the functions a cell is currently performing, the presence or absence of particular gene products that mediate the turning on and off of the production or function of the gene products targeted by the regulatory action can be used to specify whether one or another particular bank of genes will be acted on and whether the action will be an induction or cessation of their action. This capacity to use a single detector of a particular environmental shift to specify differing particular responses for cell types posing distinct types or levels of risk to the organism in their reaction to a threatening environmental action or an internal malfunction is one of the ways in which cells have developed to provide the organism in which they reside with the optimal response to a particular type of damage. 

It is common for a factory, or computer system, to function on a clock, meaning that the timing of activities is quantized so that all activities are begun and completed in discrete time intervals, 0, τ, 2τ, 3τ,…. This is accomplished *via *delays that hold operations so that new operations begin only at times that are multiples of the basic period τ. For instance, think of a classical human assembly line, where each task is accomplished during a time interval *k*τ to (*k* + 1)τ. No matter when within the interval the task is completed, the next task is not begun until the time interval is completed. Another example, and one highly relevant to cells, concerns processes that require multiple inputs, for instance, the assembly of a product that requires multiple pre-assembled components. When assembly is clock-regulated, product assembly begins at a clock tick, even if all components are in place. This kind of coordination by a clock results in *synchronous* operation. A more efficient way to operate is to do away with a clock and base all activity on readiness. For instance, the next step in assembly begins with the completion of the previous step, without waiting for a clock tick. Product assembly commences once all components have arrived, excess components being held in queues awaiting their part in assembly. In a computer system, this means that instructions are executed once all necessary inputs (data and logical inputs) are ready. This kind of execute-when-ready system results in *asynchronous* operation and, in the context of computation, is sometimes referred to as a *data-flow* system. Asynchronous operation is more efficient but also more difficult to control, requiring more complex regulatory logic. Ignoring interrupts, a synchronous system can be modeled deterministically, but an asynchronous system is inherently stochastic because variability in individual operation times is not normalized by clock periods. Nevertheless, if the regulatory logic can handle an asynchronous system, there is significant gain in efficiency.

A factory requires redundancy to keep operations running smoothly. In the worst case scenario, the failure of a single operation can halt factory operations. These kinds of catastrophic failures are avoided by eliminating their points of occurrence or, perhaps more simply, building in redundant operations, for instance, back-up generators. Multiple subsystems can be employed with back-up capabilities. Optimization of redundancy is nontrivial because too much redundancy renders the overall operation too costly. Redundancy is made more efficient by using subsystems that can perform multiple tasks and therefore be able to serve as back-ups when needed. If a unit fails, the regulatory logic needs to be able to reconfigure the operations automatically to maintain productivity, albeit, perhaps at a reduced level if back-up systems are less efficient (and therefore less costly). Fault tolerance is enhanced by the ability for autonomous correction of failures, for instance, by error correction code that checks for faults and corrects these faults when discovered. Regarding the development of control systems to regulate this kind of autonomous reconfiguration, Pugachev writes,

It is also feasible to distinguish another category of control systems which are capable of analyzing their own operating conditions and using this information to produce an optimum performance. The simplest systems of this type, which incorporate elements for automatically adjusting particular parameters according to an analysis of input and output data, are called *self-adjusting* systems. Complex systems of this kind are capable of adapting themselves completely at each instant to the results of their analysis of external conditions and previous performance. These are said to be *self-organizing*. It is quite clear that no theory of error under average operating conditions is adequate for the design of self-adjusting and self-organizing systems. A special theory is required which will solve the complex problems involved in processing the input data and utilizing it to best advantage in any particular case. Both problems can be tackled by the modern theory of optimal systems [7].

Self-organization is more than simple redundancy. It allows a system to reconfigure itself to achieve optimal (practically, close to optimal) performance under varying conditions.

A fundamental way to achieve redundancy, as well as efficiency, is through the use of parallelism. Parallel assembly of independent components is obviously beneficial, as is regulatory parallelism. If a sequence of signals must be sent to various points in the system to result in a final instruction, then fault tolerance is achieved by sending multiple signals through different paths. If one path is blocked, the signal will still arrive *via *another. In fact, the final instruction may be assembled from packets of code that have been sent through multiple channels with the packets including instructions on how they should be assembled at the endpoint. This approach provides both redundancy and enhanced speed of operation in cases where one channel is slowed owing to too much traffic or technical problems. In such a system, any channel or processor may be carrying or implementing many tasks simultaneously. 

Cells also use redundancy and parallelism to deal optimally with damage and malfunction. Redundancy is commonly observed in a cell’s response to ionizing radiation. One gene involved in a wide variety of stress related responses is tumor protein 53 (TP53). TP53 serves as a central hub in the network of stress response and it can activate an array of responses, yet it is not always required for the occurrence of such responses. Many stress response processes can be successfully mounted even in the absences of this protein, even though when TP53 is present it drives that particular process. In these cases, other proteins have been identified that are competent to drive the response in TP53’s absence. Such redundancy offers a sound way for cells to minimize the risk of failure of a critical function.

To study regulation in metabolic processes, the appropriate experimental designs and analyses will necessarily differ from the designs and analyses used for examining regulation in stress response. In shifting from regulatory relationships that are simple, linear, context-independent, and not highly branched to those that are complex, nonlinear, context-dependent, redundantly represented and both highly branched and interpenetrating, one must take into account the vastly increased number of ways the process of interest can be configured. Consider the consequences of carrying out a stress response study where the experimental plan is based on linear expectations, such as challenging a cell line with wild-type TP53 and a mutated derivative of that cell line not producing functional TP53, and then determining which genes are induced by radiation in the TP53^wt^ line that were not induced in the TP53^mut^ line. If one were to interpret these results as indicating that only those genes induced in the TP53^wt ^line and not in the TP53^mut^ line are normally dependent on TP53^wt^, one would be substantially in error. The numerous TP53^wt^ responses that can be induced independently of TP53^wt^ through redundant mechanisms would be incorrectly considered to be not normally dependent on TP53^wt^. Confronting this problem requires one to envision a different type of network architecture, where the possible antecedents of a step in a pathway are multiple and produce the same outcome. One way to do so is to formulate a test that asks what happens when the gene of interest is active. If the results in that case are in agreement with the results when it is inactive, it must be considered as a possible controlling gene for which there is a redundant controller. This obviously is not definitive; however, it will identify realistic possibilities that would not even be considered by an analysis that makes linear assumptions.

Closely related to parallelism is locality: operating decisions should, wherever possible, be made at the local level. This means that control is distributed throughout the factory. Hierarchical control suffers from at least three serious flaws. First, it is unable to efficiently respond to changed conditions at the local level. If a machine is beginning to operate unsatisfactorily, perhaps needing overhaul or replacement, this is seen immediately at the local level and, presuming the ability to rectify the situation exists at the local level, is most efficiently handled there, or as close to the operational location as possible to maintain overall functioning of the system. If a long chain of command is required to make the decision as to how to proceed, this takes time and can lead to a delay in decision-making, and thus a consequent down time. A second problem with hierarchical control is fragility. The longer the chain of command, the more likely it will be broken along the way and no decision be forthcoming – in an extreme case, the center of the hierarchical regulatory system might fail, thereby brining the entire factory to a stop. Finally, a long hierarchical chain can result in the decision resting in the hands of a decision-maker less qualified relative to the specific machine. 

As stated in our discussion about metabolism, the functions most commonly shared and heavily engineered by selection, have extremely local regulation of activity. In many cases, the enzymes that carry out a particular function are autoregulatory, having interactions with the metabolite that they produce that let them adjust their level of catalytic activity based on the local abundance of their product. The fineness of this control is sufficient to produce both high levels of rapid adaptability to fluctuations anywhere in the network of operations and a level of stability that centrally driven regulation cannot achieve.

When observing a factory involving many subsystems, the high dimensionality of the operation is typically apparent, but what might be overlooked by the casual observer is the multivariate character of the individual decisions or operations within the overall structure. Multiple inputs are often required before execution. It is not simply that multiple components are required to execute a specific assembly; more significantly, multiple signals are required for a regulatory decision. For instance, there may be numerous sensors detecting changes in performance at various points and a decision to check a unit or pull it out of service may depend on multiple sensory signals. So too might a decision to override the standard control within some part of the factory and change to some specialized logic, for instance, to deal with an interrupt. The incoming signals may be quantized to binary form, so that binary logic is used to evaluate the multivariate information and make a binary decision as to whether action is to be taken. In the case of response to a potentially catastrophic interrupt, information is directed into some control point whose default value is 0 but whose value changes to 1 to canalize part of the system into a reconfigured state of operational control until the threat has passed. A cursory view of control points within the factory might reveal a sequence of changes, thereby giving the impression that the behavior of such a pathway might reveal the regulatory logic; however, although each point in this pathway might influence its successor, it is highly likely that each point is influenced by multiple signals and that the pathway only represents a trace of this activity along a certain set of points, not a dynamical trajectory in the full state space. In this sense, such a pathway represents marginal knowledge of the operations.

Similar difficulties in ascertainment are encountered in biology. In cells, not only are there multiple inputs involved in a decision, there are alterations in the hardware components that interpret the inputs, making the responses context-sensitive. In these cases, in addition to having multiple controller genes direct the same operation, a controller gene will now provoke a particular response only part of the time. This occurs when a controlling gene is capable of acting to produce a certain set of regulatory results only when its actions are interpreted by a particular set of gene products that are variably present. If one examines a set of samples in which such variable interpretation is acting, using an analytical approach that measures correlation of gene transcription on the assumption that a gene exerting a controlling effect on a target gene should show strong correlation in expression activity, then the context-dependent controller will likely be overlooked. Its correlation with its contextually controlled targets is only evident when the controller is in the proper context. This is a widespread problem in gene control, since in the cell, every gene being expressed is regulated by other genes and frequently there are multiple regulatory conditions for a gene to be expressed, so that any set of samples is likely to have many genes being controlled by different genes in different samples, thereby making simple correlation a poor way to identify regulatory connections. 

While biologists are aware of the variety and frequency of both multiplicity of control and redundancy of control, the extent to which these and other deviations from simple linearity of control confound analysis apparently has yet to have a determining influence on how biological systems are analyzed.

Once a factory exceeds a very small number of interconnected components, coordinating its operations goes beyond a commonsense, nonmathematical approach. Reminded of Galileo’s disparagement of words as constituting knowledge in the case of gravity, the situation with a complex system is orders of magnitude more resistant to everyday language and intuition. There are two basic operational issues concerning a factory: characterization and control of its operations. First, we want to characterize the input and output of the factory; second, we want to organize the operations so as to achieve optimal (or at least satisfactory) performance. Both characterization and control require a suitable conceptualization of the factory. Such a conceptualization must be mathematical for two reasons. First, characterization and control involve relations among the components and mathematics provides a relational language, and second, mathematics provides a language in which complexity can be represented in such a way as to be amenable to human analysis. Moreover, not only are complex systems beyond ordinary intelligibility and intuition – indeed, their performance is often highly counter-intuitive – but they typically cannot be fully represented mathematically because there are too many relations and, even should one achieve a very precise and highly involved mathematical description, it may well be intractable relative to solutions of the problems of interest, such as optimizing some set of relations within the system. Hence, rather than completely characterize system outputs in terms of system inputs, we satisfy ourselves with characterizing properties of the output in relation to certain properties of the input. In such typical situations we try to select variables on the input side that have big impact on important variables on the output side. 

Given that biology appears to be solving its control problems using the same sorts of approaches but doing so in a much more complex environment, it would be reasonable to assume that progress in biological science will require adopting the same stance as engineering has already found useful, and necessary, in dealing with complexity – with the constraints being even more demanding in biology owing to a much greater degree of complexity. Hence, research must focus on finding levels of operation of biological control where prediction based on some input variables produces useful levels of prediction on the output variables.

Just as a factory’s constituent parts – electrical, mechanical, and chemical – are required for the factory to exist, the physical-chemical constituent parts of a cell are required for the cell to exist. Moreover, just as the constituent parts of a factory do not constitute the factory, but rather the regulatory (operational) logic of the factory defines the factory as an operational system whose purpose is to consume energy, maintain itself, and produce an output, the constituent parts of a cell do not constitute the cell, but rather the regulatory (operational) logic of the cell defines the cell as an operational system whose purpose is to consume energy, maintain itself, and propagate. For both factory and cell, the regulatory logic determines the relations between the physical structures within the system and between the system and its environment. By regulatory logic we do not simply refer to simple binary deterministic logic but to mathematical functions, perhaps binary in nature, that provide operational control within the framework of random processes. The roles of regulatory logic in the factory (or complex machine) and the cell are congruent because the key to the characterization of this logic lies in communication (between components) and control (of components) – that is, in systems theory, which therefore determines the epistemology of the cell. 

To illustrate key epistemological points, we consider a regulatory model that incorporates several of these points, including latency, context-dependence, distributed regulation, multivariate gene interaction, and stochasticity [25]. Because regulation is parallel and distributed, if one views the cascade of activities resulting from the action of a single regulatory gene, both the strength and specificity of subsequent activities in the cascade may be expected to diffuse through subsequent steps in the cascade. As the regulatory effects propagate, they are progressively modified or limited by interactions with other factors modulating gene transcription. 

One can view genes at various positions in a regulatory cascade as being either masters or slaves, keeping in mind that this is a relative characterization and that in certain situations a gene might act as a master, while in others in might act as a slave. If the situation were one of strict, complete control of one gene by another at all times, then gene *g* being a master of gene *g*_1_ in a binary *ON-OFF* genetic regulatory model would mean that *g* → *ON *implies *g*_1_ → *ON*, and that *g* → *OFF* implies *g*_1_ → *OFF*. This kind of strict control is not indicative of distributed regulation; indeed, in a distributed environment, *g* → *ON *would not necessarily imply *g*_1_ → *ON*, since *g* may only be able to set *g*_1_ → *ON* in coordination with other genes.

To illustrate the resulting context-dependent behavior of a model system, suppose genes *g*_1_ and *g*_2_ are fully controlled by genes *g*, *g*_0_, and *g*_00_ (which may be in turn regulated, or affected, by other genes in any number of cascades). Table **1** shows a possible regulatory structure for five genes and Fig. (**1**) shows a network diagram consistent with this structure. Genes *g*_0_ and *g*_00_ are not part of the model; however, their states are physically co-determinative along with the model master *g* of the model slaves *g*_1_ and *g*_2_. The four possible combinations of the states of *g*_0_ and *g*_00_ determine four possible contexts, *C*_1_, *C*_2_, C_3_, and *C*_4_, for the model. Given the context, the relationship of the state of *g* to that of its slaves is determinative; however, absent knowledge of the context, it is not. If *g*_1_ = 1 and *g*_2_ = 1 in context *C*_1_, then *g* = 1; if *g*_1_ = 1 and *g*_2_ = 1 in context *C*_4_, then *g* = 0. It cannot be that *g*_1_ = 1 and *g*_2_ = 1 in contexts *C*_2_ and *C*_3_.

Conceptually, the regulatory action within the model is viewed as a system with inputs corresponding to the regulating master genes for the slave genes; however, the system is not fully described by the input gene values alone, but by these inputs in conjunction with the context. Biologically, the context is determined by the manner in which the slaves are responding to latent genes external to the model network. Together, the latent genes act in a manner as to select a network (system) context. One can imagine a set of input lines entering the overall system, a family of subsystems (contexts) within the system, and the system output being a single line whose information is selected from among the subsystems. This would be the structure of a computer system whose output is determined by a multiplexer, with the multiplexer’s decision being determined by a selection input to it. The model system behaves deterministically so long as it remains in a fixed context.

We now describe the master-slave model [25], restricting ourselves to a single master gene *g* and a corresponding set *S* = {*g*_1_, *g*_2_, ..., *g_r_*} of slaves (see [25] for a more general formulation). The genes in *S* may be influenced by genes other than *g*. Let *Y* be the binary expression value for *g* and **X **= (*X*_1_, *X*_2_, ..., *X_r_*) be the binary-valued expression vector for the slaves. Control by *g* is of the following form: if *Y* = 1, then all genes in *S* take on the value 1 with high probability. We let *p* = *P*(*Y* = 1).

If *Y* = 1, even though the master is *ON*, contextdependent regulation may affect the slaves. For any slave $g_{k}\in S$ , the conditional probability of *g_k_* being *ON* is given by

$P\left(X_{k}=1|Y=1\right)=1-\delta_{k}$

where the magnitude of δ_k_ depends on the extent to which the influence of the master on *g_k_* is diminished by contextual effects. To illustrate the meaning of this conditional probability, consider Table **1**. Partitioning the probability according to the contexts yields 

$P\left(X_{1}=1|Y=1\right)=\frac{\sum_{j=1}^{4}P(Y=X_{1}=1|C_{j})P(C_{j})}{\sum_{j=1}^{4}P\left(Y=1|C_{j}\right)P\left(C_{j}\right)},$

where *P*(*C_j_*) is the probability of the context *C_j_*. The size of δ_1_ depends on the conditioning of the contexts and their probabilities. Suppose contexts *C*_2_ and *C*_4_ cannot occur, so that *P*(*C*_2_) = *P*(*C*_4_) = 0. Table **1** shows that

$P\left(Y=X_{1}=1|C_{1}\right)=P\left(Y=1|C_{1}\right)$

and

$P\left(Y=X_{1}=1|C_{3}\right)=P\left(Y=1|C_{3}\right).$

Thus, *P*(*X*_1_ =1 | *Y* = 1) = 1 and  δ_1_ = 0. Conditioning with the control that *X*_1_ = 1 when *Y* = 1 occurs due to contexts *C*_2_ and *C*_4_, so that if they do not occur, there is no such conditioning. A similar analysis applies to *P*(*X*_2_ = 1 | *Y* = 1), and in this case conditioning with the control that *X*_2_ = 1 when *Y *= 1 occurs due to contexts *C*_3_ and *C*_4_. We refer to δ_*k*_ as the *conditioning* parameter. 

If *Y* = 0, then the probability that *X_k_* = 1 depends on contextual effects when the master is not actively regulating the slaves. We let

$P\left(X_{k}=1|Y=0\right)=\delta_{k}.$

From Table **1**, partitioning the probability according to the contexts yields

$P\left(X_{1}=1|Y=0\right)=\frac{\sum_{j=1}^{4}P\left(Y=0,X_{1}=1|C_{j}\right)P\left(C_{j}\right)}{\sum_{j=1}^{4}P\left(Y=0|C_{j}\right)P\left(C_{j}\right)}.$

Again suppose contexts *C*_2_ and *C*_4_ cannot occur. From Table **1**, we see that 

$P\left(Y=0,X_{1}=1|C_{1}\right)=P\left(Y=0,X_{1}=1|C_{3}\right)=0,$

so that *P*(*X*_1_ = 1 | *Y* = 1) = 0 and η*_k_* = 0. A positive value of η*_k_* means that it can be that *X*_1_ = 1 absent the forcing control of the master when *Y* = 1. A similar analysis applies to *P*(*X*_2_ = 1 | *Y* = 0). We refer to η*_k_* as the *crosstalk* parameter because genes outside the model are turning the slaves on.

The model is determined by the two conditional probabilities defining the conditioning and crosstalk parameters. They characterize our understanding of regulation in the model. If there is very little conditioning and little crosstalk, then η*_k_*  is substantially smaller than 1 − δ_*k*_.

Crosstalk poses implications for experimental design. Suppose one takes a large number of samples over unknown contexts. It may be that a master exhibits tight control (perhaps with no external conditioning) across all study samples, but when that master is not on, the behavior of the slaves is controlled by other genes. If under this other control the slaves are uniformly distributed *ON* and *OFF*, we have the situation δ_*k*_ = 0.5. If the probability mass of the contexts in which *g* → *OFF* greatly outweighs the mass of those contexts for which *g* → *ON*, then the determinative effect of *g* on the slaves can be very low across the study samples. Essentially, the experimenter is blinded. Even worse, the experimenter can be severely fooled. If the slaves are mostly *OFF* outside the control of the master, so that the crosstalk parameter is very small, even if the master exhibits little control when it is *ON*, it might well show a stronger determinative effect than a master that exhibits tight control but has slaves that respond significantly to experimental conditions outside the study examples for which the master is *ON*. 

## CONCLUSION

Biology studies relations between molecules (chemical structures), not the molecules or the forces between molecules. The recognition that biological knowledge concerns regulatory logic and the consequent intra-cell operational organization of molecular structures, as well as, by extension, inter-cell organization, entails the concomitant recognition that biological systems, in their extraordinary complexity, are beyond everyday intelligibility and intuition. Moreover, it facilitates answers to the two fundamental epistemological questions raised in the Introduction: (1) What form does biological knowledge take? (2) How is biological knowledge validated? The question as to form relates to the type of mathematics involved in modeling the relations that characterize regulatory knowledge. This depends on the nature of the relations being considered; however, the general mathematical framework will be formed within the theory of stochastic multivariate dynamical processes. Validation depends on the mathematical model characterizing the knowledge and, since this knowledge concerns operational regulation, validation will involve operational predictions derived from the mathematical model.

## Figures and Tables

**Fig. (1):**
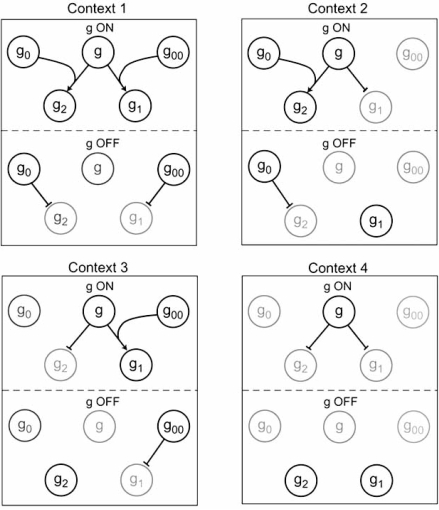
Regulatory inputs from genes g, g_0_, and g_00_ on genes g_1_ and g_2_ for four contexts.

**Table 1:** Truth Table Showing the Consequences of Regulatory Inputs from Genes g, g_0_, and g_00_ on Genes g_1_ and g_2_

Contexts	*g*_0_	*g*_00_	*g*	*g*_1_	*g*_2_
*C*_1_	1	1	1	1	1
	1	1	1	1	1
*C*_2_	1	1	1	1	1
	1	0	0	1	0
*C*_3_	0	1	1	1	0
	0	1	0	0	1
*C*_4_	0	0	1	0	0
	0	0	0	1	0
